# Integrating theory and empirical patterns: Fish body size distributions, life history traits, and environmental flows in streams

**DOI:** 10.1126/sciadv.adu4026

**Published:** 2025-12-19

**Authors:** Taylor Woods, Daniel J. McGarvey, Matthew J. Cashman, Michael R. Meador, Daren M. Carlisle, Ken Eng, Darin A. Kopp, Kelly O. Maloney

**Affiliations:** ^1^ Eastern Ecological Science Center, US Geological Survey, Kearneysville, WV, USA.; ^2^ Center for Environmental Studies, Virginia Commonwealth University, Richmond, VA, USA.; ^3^ Water Mission Area, US Geological Survey, Baltimore, MD, USA.; ^4^ Water Mission Area, US Geological Survey, Reston, VA, USA.; ^5^ Water Mission Area, US Geological Survey, Lawrence, KS, USA.; ^6^ Office of Research and Development, US Environmental Protection Agency, Corvallis, OR, USA.

## Abstract

Individual size distributions (ISDs) are prominent in ecological research and may support resource managers with ecosystem-scale objectives. We use a database of individual size measurements for US stream fishes to test for direct and indirect effects of traits, flow regimes, and land use on the interspecific ISD exponent. Path analysis indicates that traits have strong, direct effects on ISD. Flow and land use effects on the exponent are largely indirectly mediated by their influences on species traits. ISD exponents increase (abundances of larger-bodied individuals increase, relative to smaller-bodied) when environments favor higher trophic levels, warmer thermal tolerances, and periodic life histories. Alternatively, ISD exponents decrease in systems that favor opportunistic life histories. Our flexible modeling framework that includes direct and indirect effects of traits, flow regimes, and land use on ISD could be expanded to incorporate additional variables that interact with flow (e.g., temperature and physical habitat) to assess of effects of multiple stressors on aquatic ecosystem functioning.

## Teaser

Environmental flow relationships help predict ecosystem scale effects of flow regime alteration.

## INTRODUCTION

Body size plays a vital role in ecology. It is fundamental to individual metabolism and growth and, by extension, population density and production (*1*, *2*). Body size influences dispersal capability and range size (*3*, *4*), elemental stoichiometry, and nutrient cycling rates (*5*–*7*). It also regulates predator-prey dynamics and is therefore a central determinant of food-web structure and stability (*8*, *9*). For these reasons, statistical distributions that describe body sizes across individuals or species are essential to ecological research (*10*).

At the assemblage level, body size and abundance follow an inverse power law distribution, with many small and relatively few large individuals (*11*). Models of this individual size distribution (ISD), or “size spectra,” use ataxic data where individuals within assemblages are identified solely by their size, irrespective of taxonomic identity (*12*). This approach is intuitive in aquatic systems where many species experience ontogenetic shifts in critical, size-dependent behaviors, such as predation, or exhibit distinct variation in adult body size within and among populations (*13*–*15*). At a given moment or location, individual size may predict behavior more accurately than taxonomy (*14*, *15*).

Exponents (*b*) of power law ISD models are particularly interesting because they facilitate comparisons of the relative abundances of small-to-large organisms through time and among systems (*14*, *16*). For example, *b* was shown to decrease (i.e., disproportionate decline in the abundance of large individuals, relative to smaller individuals) in aquatic systems as ambient water temperature increased (*17*). Similarly, *b* decreased in Celtic Sea and coral reef fish assemblages as fishing pressure selectively depleted the largest fishes (*18*, *19*). These changes in *b* can indicate altered trophic transfer efficiency (TTE) and energy flow through food webs from changing environmental conditions or anthropogenic influences (*20*). ISD research may therefore benefit management efforts that consider ecosystem-level objectives, such as mitigating the effects of invasive fishes on stream food webs (*21*).

Building on historical research in marine systems (*13*), ISD studies have proliferated in the freshwater fish literature (fig. S2). Recent examples include *b* responses to nutrient pollution and non-native fishes in Iberian streams (*22*) and to allochthonous subsidies in trout streams throughout the United Kingdom (*23*). In large, regulated rivers, ISD research has demonstrated a meaningful influence of hydropeaking on *b* (*24*), as well as gradual shifts in food-web structure and ISD *b* through time (*25*).

The increased rate of ISD publications, notably, has coincided with accelerating rates for two other freshwater topics: traits-based research and effects of flow regimes (fig. S2). Functional traits are the physiological, reproductive, and trophic characteristics that define species roles, or niches, within ecosystems (*26*). In streams, four dimensions of the flow regime—flow magnitude, duration, frequency, and timing of high (flooding) and low (drought) events—comprise an essential physical template that species traits are adapted to (*27*). Now, evidence of predictable relationships between species traits and flow regimes (hereafter “environmental flow” relationships) is accumulating across broad spatial scales. This work includes, but is not exclusive to, trophic (*28*), reproductive (*29*, *30*), and physiological tolerance (*31*, *32*) traits.

In this study, we merge ISD, functional trait, and environmental flow research within a single modeling framework. From an empirical and practical standpoint, the required body size, trait, and flow data to build and test models at regional to national scales have recently become available (*33*, *34*). This integration may help fill a key knowledge gap because the effects of flow on the ISD have received less attention than other factors, such as, temperature, water quality, and land use (*17*, *22*, *35*). Engineered flow regimes can also be important management tools. Hence, studies that integrate environmental flows, traits, and the ISD may guide ecosystem-scale efforts to restore healthy flows in flow-altered systems (*36*).

Ecological theory also suggests that the integration of ISD, functional traits, and environmental flow research may reveal additional insights. The trilateral continuum of fish life history posits that reproductive traits are adapted to flow regimes that regulate spawning, juvenile survival, and recruitment success (*29*, *30*). For example, opportunistic strategists depend on small clutches (limited number of eggs), serial spawning events, and rapid maturation rates that may ensure that some offspring will survive and reproduce in harsh, stochastic environments (*37*). Because short generation times also mean that most opportunistic strategists have small adult body sizes, *b* may decrease when the prevalence of small, opportunistic species is high. Alternatively, fishes with periodic life histories are adapted to seasonal flow regimes where the timing of high-flow events is predictable (*38*, *39*). These iteroparous species can delay spawning events for extended periods and then quickly deposit large numbers of eggs when conditions become favorable. ISD *b* is therefore likely to increase with the prevalence of relatively large periodic fishes.

Functional trait theory may also help to explain associations between flow regimes and the ISD. For instance, trophic position not only often increases with body size in temperate freshwater systems (*40*, *41*) but also is influenced by the flow regime (*31*, *42*). These observations suggest that *b* might decrease when altered flow regimes prove unfavorable to large, piscivorous fishes. Thermoregulatory capacity may also link flow regimes to the ISD. Anthropogenic flow reductions (e.g., agricultural and water withdrawals) can increase water temperatures, particularly in warmer summer months (*43*). These warmer temperatures may decrease *b* because small-bodied ectotherms are generally more tolerant of high ambient temperatures than larger ones (*44*).

**Fig. 1.**
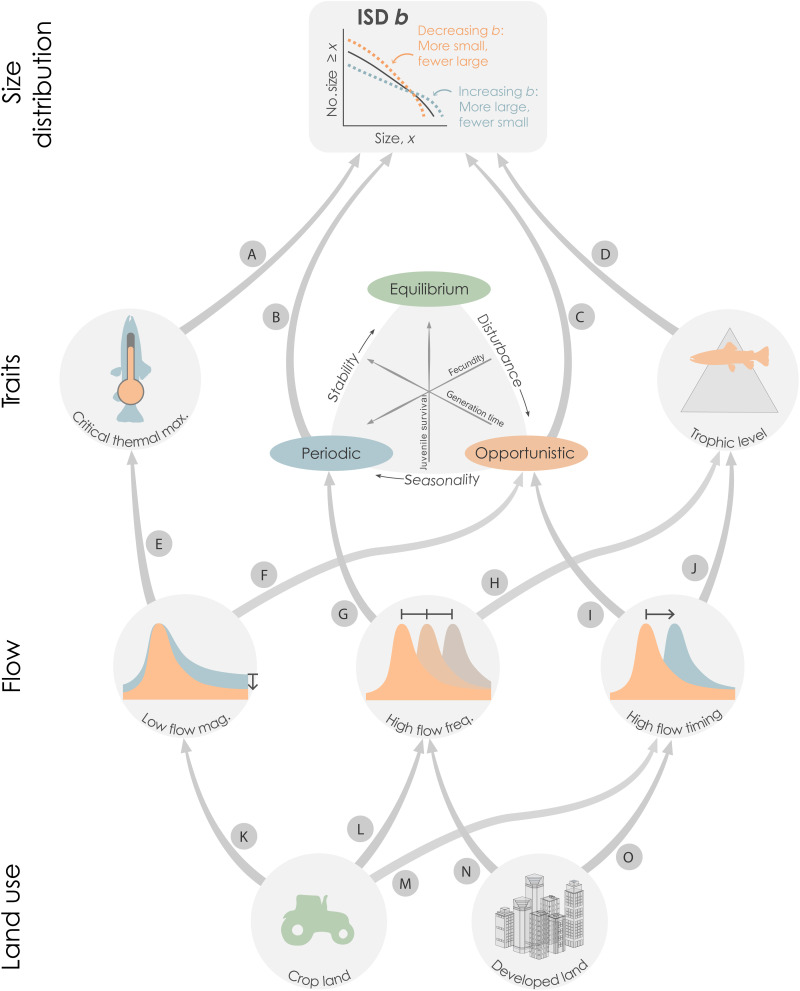
Meta-model illustrating hypothesized links between ISD *b*, fish traits, flow, and land use. Labels on individual paths correspond to hypotheses explained in Materials and Methods and table S1. The second layer (“traits”) shows potential linkages between critical thermal maximum, life history (periodic and opportunistic affinities), and trophic level on ISD *b*. The third layer (“flow”) visualizes linkages between flow alteration metrics of low-flow magnitude, high-flow frequency, and high-flow timing. Flow alteration metrics are visualized using multiple distributions that indicate potential expected and observed distributions (indicated by colors), with arrows indicating the potential shift in directionality from expected to observed. The final layer (“land use”) visualizes potential linkages between crop and developed land uses on flow alteration metrics.

Guided by the above logic, we begin this study with a sequential, two-stage hypothesis linking traits and the flow regime to *b*. We expect that life history and functional traits will have strong, direct effects on ISD *b*, whereas flow regime effects on *b* will be indirect and mediated by their direct effects on traits. Using the notation of a path analysis, this simple hypothesis can be written as flow → traits → *b*. Next, we extend this hypothesis to a literature-informed meta-model (*45*), where *b* is directly influenced by two functional traits (species’ critical thermal maximum and trophic level; Fig. 1, A and D) and two life history traits (prevalence of periodic and opportunistic species; Fig. 1, B and C). Functional and life history traits are, in turn, influenced by variation in three environmental flow parameters (low-flow magnitude, high-flow frequency, and high-flow timing; Fig. 1, E to J, and refer to Materials and Methods). Last, flow is linked to two exogenous land use variables (percent crop cover and percent developed land; Fig. 1, K to O; specific hypotheses are provided in table S1 and described in Materials and Methods).

To test the meta-model, we use a large collection of assemblage-level fish samples, including individual size measurements, from streams throughout the conterminous US (CONUS). For each fish assemblage, maximum likelihood is used to estimate *b* (*16*, *46*). Species traits, including primary life history strategy, trophic level, and critical thermal maximum, are appended from published sources (*33*, *47*, *48*). Flow alteration metrics that account for deviations from natural flow regimes (i.e., in the absence of anthropogenic disturbances) are used to quantify alterations to the magnitude, duration, frequency, and timing of high- and low-flow events, providing links to fish life history and functional trait theory (*37*). A formal model selection (*49*) and path analysis (*50*) process is then used to test the meta-model, resulting in a quantitative, systems-level integration of life history, functional trait, environmental flow, and ISD theory.

## RESULTS AND DISCUSSION

**Fig. 2.**
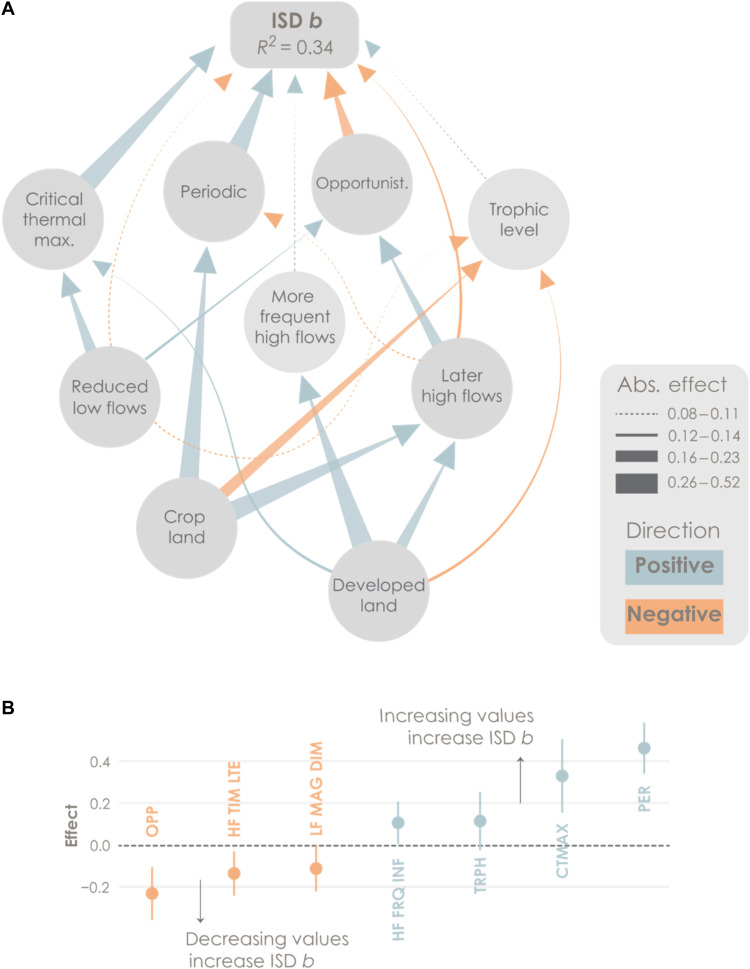
Path analysis results. (**A**) Final path model of the ISD exponent (*b*). Arrow colors distinguish positive negative effects. Arrow stroke width increases with effect size [absolute (abs.) value of the path coefficient]. Fixed effects of natural control variables (drainage area and ecoregion) are not shown in the path diagram. (**B**) Fixed-effect sizes (with 95% confidence intervals) for direct predictors of ISD *b* abbreviated as follows: OPP, opportunistic affinity; HF TIM LTE, later high flows; LF MAG DIM, diminished low-flow magnitudes; HF FRQ INF, more frequent high flows; TRPH, trophic level; CTMAX, critical thermal maximum; PER, periodic affinity.

The compiled data included 272 independent, assemblage-level estimates of *b* (mean = −1.43, SD = 0.44). A final path model that included each of the nine variables in Fig. 1 as fixed effects fit the data well (Fisher’s *C* = 37.14, *P* = 0.68) and accounted for 34% of the variation in *b* (Fig. 2). Drainage area and ecoregion were included as fixed effects in the final path model to account for natural variation in stream/river size and turnover in the composition of regional species pools, respectively. Traits consistently had the strongest direct effects on *b*, with positive effects of periodic strategy affinity (calculated as the assemblage weighted mean; refer to Materials and Methods), critical thermal maximum, and trophic level but negative effects of opportunistic affinity. Flow variables had mixed effects on *b*. Delayed (later) high-flow timing had a direct, negative effect on *b*, in addition to indirect effects mediated by periodic and opportunistic life history strategies. Diminished (lower) low-flow magnitude had a direct, although weak, negative effect on *b*, in addition to indirect effects mediated by opportunistic affinity, critical thermal maximum, and trophic level. More frequent high-flow events directly increased *b*. Crop land use and developed land use effects on *b* were mediated entirely through altered flows and traits.

### Direct trait effects on ISD *b*

Path analysis confirmed our initial hypothesis that fish species traits would have stronger direct effects on the ISD than flow or land use (Fig. 1). Each of the four modeled traits was a significant predictor of *b* (*P* < 0.05). Traits also accounted for the three largest effect sizes among all direct, fixed effects on *b* (Fig. 2). Of these, life history traits stood out as important. The periodic and opportunistic affinities had strong effects that supported predictions: *b* increased (relative abundance of large individuals increased) with the prevalence of large, highly fecund periodic species but decreased with the prevalence of small, rapidly maturing opportunistic species. To our knowledge, this study is the first to demonstrate an explicit link between Winemiller’s (*37*) seminal “triangular” life history model and the ISD. Others have used variants of traditional bioenergetics models to simulate effects of variable life history traits on the ISD but stopped short of using empirical data to validate those models (*51*, *52*). We used empirical fish assemblage data to show that life history traits directly influence *b*, expanding upon ISD theory (*53*).

These trait and flow regime insights can also guide management decisions. For example, flow regimes can be engineered to mimic seasonal environments that favor large-bodied, periodic species (*54*). This strategy was applied in Putah Creek, CA when a regulated flow regime, featuring stable summer baseflow bracketed by spring and winter pulses (providing spawning and migration cues and juvenile rearing habitat), was used to restore the native fish assemblage (*55*). Alternatively, Novak *et al.* (*21*) used the ISD to estimate preinvasion conditions and to set specific restoration goals for an invasive species removal and native biomass recovery project. Because regulated rivers are so prevalent, the integration of flow, trait, and ISD knowledge may have potential to assist in aquatic conservation and restoration efforts.

The predicted positive effect of trophic level on *b* was also confirmed, although weaker than the other trait effects (Fig. 2). This deviated from our initial hypothesis that an increasing number of large predators would strongly influence *b*. In retrospect, we see two potential flaws in our original logic. First, the requisite assumption of a robust positive relationship between trophic level and body size may be incorrect. Although some studies report positive size versus trophic level relationships (*31*, *32*), others report conditional or nonexistent relationships (*56*, *57*) or challenge the underlying assumption (*58*). Second, the connection between body size, trophic level, and *b* may be too complex to model with simple linear relationships. A commonly applied model of *b* includes the predator-prey mass ratio (PPMR), TTE, a scaling constant (λ) and takes the form: ISD *b* = λ + log(TTE)/log(PPMR), where canonical values of PPMR = 10,000, TTE = 0.10, and λ = −0.75 are often used as initial parameter estimates (*23*). This model suggests that size and trophic level, each of which is implicit in the TTE and/or PPMR terms, share a dynamic and potentially compensatory effect on *b*. If correct, then a direct effect of trophic level on *b* is less likely, and PPMR estimates may prove more useful than trophic level to model *b*.

Critical thermal maximum also had a strong effect on *b*, although the positive direction (Fig. 2) was opposite our original prediction. Because higher thermal tolerances are often observed in smaller fishes (*44*), we anticipated a negative relationship with *b*. This unexpected result has challenged us to rethink the complex dynamic between body size, growth rate, thermal tolerance, and environmental temperature. For instance, warmer environments might increase growth rates or extend the growing season resulting in larger body sizes, even for cold-water fishes (*59*). However, the positive link between thermal tolerance and *b* may also reflect covariance between critical thermal maxima and a trait that was not included in our analysis. For example, generalist life history strategies may facilitate the growth and spread of non-native fishes with large bodies and relatively high thermal tolerances, such as invasive grass carp (*Ctenopharyngodon idella*) or silver carp (*Hypophthalmichthys molitrix*) (*60*). Regardless, additional research on the connections between environmental temperature and species body sizes and thermal tolerances is needed to protect ecosystem function in the face of rising temperatures.

### Mixed effects of flow

**Fig. 3.**
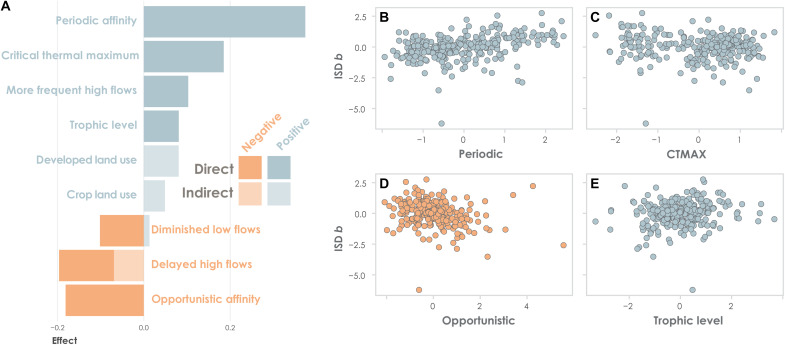
Direct and indirect effects on ISD *b*. (**A**) Total (direct + indirect) effect sizes are partitioned into direct (solid bars) and indirect (transparent bars) effects for all fixed effects in the ISD *b* model. Bar colors distinguish positive (blue) from negative (orange) effects. Variables are defined in Fig. 2 (main text). (**B** to **E**) Relationships between ISD *b* and traits including (B) periodic affinity, (C) critical thermal maximum, (D) opportunistic affinity, and (E) trophic level.

As expected, flow effects on ISD *b* were often indirect and mediated by traits (Fig. 2). However, each flow variable also had a direct effect on *b* that we did not anticipate. Combined direct and indirect effects of high-flow timing were particularly strong (Fig. 3A). The predicted, positive effect of delayed high flows on opportunistic affinity was confirmed (Fig. 1 and table S1), resulting in a net negative effect on *b*. However, the negative effect of delayed high flows on *b* was further strengthened by two pathways that we did not originally consider: via a direct, negative link to *b* and by constraining the positive effect of the periodic affinity on *b*. High-flow events can influence temperate fish assemblages in numerous ways. Depending on timing, high flows may serve as spawning and migration cues. They may also have short-term disruptive effects, such as scouring eggs or displacing juveniles, or long-term effects on physical habitat and geomorphology (*27*, *61*). At present, our ability to model habitat effects is constrained by a lack of paired habitat data. Adding data and model paths to test habitat-mediated effects of flow on functional groups and spawning guilds may help further clarify influences of altered flow timing on *b*.

We predicted that altered high-flow timing would favor small-bodied opportunistic fishes. These species use classic *r*-selected strategies (fast life cycles, multiple clutches, and minimal parental care) and do not rely on seasonal flows to initiate reproduction (*39*). Hence, they may be less sensitive to altered high-flow timing (*30*, *62*). We did not, however, predict the observed negative effect of delayed high flows on large periodic species (Fig. 2). This was an oversight as large fishes often depend on seasonal high flow cues to complete their life cycle and may therefore be sensitive to altered high-flow timing (*63*). Last, we acknowledge the direct, negative path between delayed high flows and *b*, which was not predicted in the meta-model (Fig. 1), may reflect any number of plausible mechanisms, such as selective displacement of large benthic prey during the critical summer/fall growth period (*64*). In summary, our results indicate that high-flow timing has multiple direct and indirect influences on the size structure of temperate fish assemblages, which may be investigated in future research.

An overall negative, multipath effect on *b* was also observed for diminished low flows (Fig. 2). As predicted in the meta-model (Fig. 1), both the opportunistic affinity and critical thermal maximum traits increased as low-flow magnitude decreased. This may indicate a disruptive effect of drought conditions on aquatic habitat that may benefit species with flexible, opportunistic life histories (*30*). However, we did not predict the direct negative effect of low-flow magnitude on *b* or its moderating effect on trophic level. The direct link may indicate an acute habitat effect that is independent of species’ life history strategies. For instance, habitat loss through drying events can create isolated, shallow pools where larger fishes are disproportionately vulnerable to oxygen depletion, elevated temperature, and terrestrial predators (*65*, *66*). A similar process could explain the negative link with trophic level. If a positive relationship between body size and trophic level exists (*40*, *41*), then selective survival of smaller fishes would result in a lower average trophic level under low-flow conditions. These low-flow insights are important because drought is expected to increase throughout much of the CONUS (*67*). For the moment, we emphasize that the net negative effect of diminished low flow on *b* was evident, despite the offset, mediated effect of critical thermal maximum (Fig. 3A), and conclude that low-flow magnitude is, similar to high-flow timing, a key determinant of size structure.

Streams with more frequent high flows had higher *b* values, but the effect was direct (Fig. 2) and did not follow the predicted, mediated paths through periodic life history or trophic level traits (Fig. 1 and table S1). This was unexpected because others have reported a strong association between the prevalence of periodic fishes and the frequency or duration of high-flow events (*29*), which can regulate floodplain access and trophic subsidies (*68*). We propose that the direct link between high-flow frequency and *b* may be an outcome of selective resistance to high-flow displacement. In high-discharge events, small fishes are often displaced more quickly than larger fishes (*69*, *70*). This disparity could deplete small fish abundance, leading to increased *b*, in systems where high-flow events are repeated at rapid intervals, such as dam-regulated rivers with daily or weekly hydropeaking schedules (*71*). Rapid high-flow intervals might also explain why the predicted link between high-flow frequency and periodic life history was not detected; the floodplain connections that benefit periodic fishes occur at slower (monthly to annual) intervals.

### A broad land use context to study the ISD

We began this study by hypothesizing a hierarchical series of cause-and-effect relationships linking land use to flow, flow to traits, and traits to *b* (Fig. 1 and table S1). In the final path model (Fig. 2), land use effects were more complex than anticipated, with two main deviations from the meta-model. First, the hypothesized links between land use and flow were fully supported for developed land use (positive effects on delayed high flows and more frequent high flows) but not for crop land use. The predicted, germane effect of crop land use on flow was limited to a single, positive effect on delayed high flows. With hindsight, we recognize that the crop land use data, which encompass ~16% of all land in the CONUS (*72*), were probably too coarse to reveal multiple, independent effects on flow regimes. The general crop land use category of the National Land Cover Dataset can be further subdivided into 107 specific cultivated crops (*73*), many of which have distinct soil effects, irrigation needs, and growing seasons. Integration of more precise crop data may help refine and elucidate the relationships between crop land use, flows, and size structure.

Second, land use had direct effects on three of four traits (Fig. 2), none of which were included in the meta-model (Fig. 1). Parallel effects on the periodic (increasing with both crop and developed land uses) and trophic level (decreasing with both land uses) traits could reflect the fertilizing influence of crop and developed land uses. Nutrient runoff is often high in agricultural and urban settings, leading to rapid algal and/or phytoplankton growth (*74*) and high densities of herbivorous species (*75*, *76*). This would partially account for the direct, positive effects of crop and developed land uses on periodic affinity, as some of the most common periodic species were relatively large herbivores from the families Catostomidae, Clupeidae, and Mugilidae (average trophic levels of 3.0, 3.3, and 2.5, respectively). Similar logic may also explain the direct, negative effects of crop and developed land uses on trophic level.

Despite these unexpected deviations, we submit that our most important land use prediction was realized: We found no evidence of a direct land use effect on *b*. In the absence of direct links between land use and *b*, we can infer that the indirect positive effects of land use (Fig. 3A) were mediated primarily by flow and traits. For example, the net effect of developed land use was driven by its positive effect on high-flow frequency and critical thermal maximum (Fig. 2). Previous studies have documented but not fully explained empirical correlations between land use and *b* in freshwater ecosystems (*35*, *77*). Our analysis goes further by predicting and then testing explicit lines of cause-and-effect logic. Moreover, it demonstrates a fully quantitative framework to integrate theory and data on flow regimes, species’ traits, and the ISD.

### Opportunities for future ISD research

Our understanding of this large, spatially complex system is incomplete. The final model accounted for only 34% of the variance in *b*, and of 15 paths predicted in the meta-model (Fig. 1), only 10 were confirmed. The remaining five paths were rejected, while an additional eight paths were added through the stepwise model selection process. We acknowledge these limitations, but path models are flexible and extensible tools that can be refined in future research to incorporate additional data.

One fundamental variable that was omitted in this study is intra-annual variation in *b*. Temporal variation in the collection of field samples, or in the seasonal growth periods of local fishes may have added some bias to our results. For example, a study in Mid-Atlantic streams sampled in spring, summer, and fall, *b* values tended to increase later in the year (*78*). This increase was attributed to temperature-dependent growth: *b* values were lowest during the cold, pre-growth period in March but increased with the cumulative number of degree days. Highest *b* values occurred in August, when many fishes reached the end of the growing season and achieved their maximum age-class size. Hence, comparisons of *b* could account for temporal variation when working in seasonal, temperate systems, when data are available to assess intra-annual growth effects. Given the broad scope of the fish data (many samples collected from many sites, over many years), our ability to account for temporal or intra-annual differences in growth was limited, but we doubt that temporal variation unduly influenced our results. First, a large majority of the fish samples were collected within a reasonably narrow, summer sampling interval. Among all samples, the mean Julian sampling date was day 231 (SD = 33 days), with modest variation in sampling dates observed between eastern (mean = day 217, SD = 45 days) and western streams (mean = day 243, SD = 30 days). Considering that summer is the major period of annual growth for most North American stream fishes (*79*), fishes at many of the sampling sites within our national database likely experienced comparable growth opportunities, before their respective sampling events.

Other variables that might enhance understanding of b include physiochemical conditions, such as physical habitat (*80*), water temperature (*81*), nutrient concentrations (*82*), and contaminant loads (*83*). These physicochemical variables may further explain intermediate links among *b*, traits, flow, and land use, or they may reveal direct effects on the ISD that are independent of traits and flow. Additional flow metrics, traits (e.g., morphological characteristics) (*84*), or trophic groups (e.g., benthic macroinvertebrates) may also help clarify unexplained variation. Because stream gages are more likely to occur near population centers and in larger streams (*85*), our results may not adequately represent remote or very small headwater systems. Last, we note that traditional research on density dependence and self-thinning in fish populations (*86*) may explain biotic influences on ISD and vice versa. The opportunities to further explore biotic and abiotic influences on assemblage size structure within a systems-level framework may continue to advance our understanding of the complex dynamics that govern the ISD.

## MATERIALS AND METHODS

### A theoretical framework to link flow alteration, species traits, and ISD

We first developed a conceptual diagram, or meta-model, that linked (i) species traits to ISD, (ii) flow alteration to species traits, and (iii) land use stressors to flow alteration. Our approach stemmed from the central assumption that land use and flow alteration would indirectly affect the ISD through trait-based filtering. It also implemented a space-for-time philosophy; we focused on relationships that are likely to occur across spatial gradients, rather than through time, because our fish data did not include consistent temporally replicated samples. Notably, we omitted many other variables that might serve as intermediate links between flow and traits, such as instream morphology and physical habitat because we did not have the requisite data. Similarly, we were not able to incorporate intraspecific variation within the four trait variables because most trait datasets are currently limited to species-level, adult characteristics.

For trait to ISD links, we identified a suite of functional and life history traits that we predicted would scale with body size (refer to Fig. 1 and table S1). We hypothesized that small-bodied fishes will have higher thermal tolerances (*87*) and high affinities for opportunistic life histories but low affinities for periodic life histories (*37*) and generally occur at low trophic levels (*41*, *88*). We expected opposite relationships for large-bodied fishes.

For flow-to-trait links, we focused on relationships between flow alteration and functional and life history traits identified in the environmental flow literature. We predicted that temperature would increase in streams with lower-magnitude low flows (*43*), selecting for small-bodied species with high thermal maxima (*87*) and strong affinities for opportunistic life histories that are tolerant of warm, drought-prone conditions (*30*). Streams with more frequent high flows may have stronger lateral connectivity and were therefore predicted to support periodic life histories, which depend on floodplains for juvenile nursery habitat for juveniles (*89*) and terrestrial prey subsidies (*68*). Altered high-flow timing was expected to act as a seasonal disturbance that would favor fishes with opportunistic life histories (*39*), while disrupting the availability of relatively large prey for higher trophic level fishes (*89*).

To model land use to flow links, we selected crop and developed land uses. These land uses are major drivers of flow alteration across CONUS (*90*) and are likely associated with many instream variables that were not directly incorporated in our model, such as instream physical habitat. Dam effects were considered but ultimately excluded from path model analyses because dams are strongly correlated with upstream drainage area, which was used as a control variable in the path model (refer to the “Path analysis” section). We predicted that streams in watersheds with extensive crop land use would have reduced low-flow magnitudes, delayed high flows, and decreased high-flow frequencies, due to water storage and irrigation withdrawals (*90*). Last, we predicted that streams in more extensively developed watersheds would have more frequent high flows (flashier flow regimes) and less predictable high-flow timing (*91*).

### Fish assemblage, size, and trait data

Data to quantify fish abundances and size distributions were obtained from the US Geological Survey, National Water Quality Assessment Project (NAWQA) (*92*, *93*). Whole-assemblage (all locally occurring species) samples were collected between 1993 and 2017 from a diversity of habitats throughout the CONUS, ranging from small streams to mainstem rivers (*92*, *93*). Because the NAWQA site selection process was not based on an a priori stratified-random sampling design (*92*, *93*), we cannot assume that the collective NAWQA database is an unbiased sample of all CONUS streams and rivers. We therefore provide summary distributions of select natural and anthropogenic variables (e.g., slope, elevation, and land use) in the NAWQA samples used in this study compared to CONUS-wide distributions for the same variables (fig. S3 and table S2). In general, these comparisons suggest that our sample data are indeed representative of the larger population of CONUS streams. The one notable exception is drainage area; very small drainages (headwater streams) are underrepresented in our data.

Fish sampling protocols were habitat specific: backpack and/or towed barge electrofishing were used in wadeable streams, while boat electrofishing was used in nonwadeable streams and rivers. Captured fish were identified and enumerated, and individual length and weight measurements were collected for at least 30 individuals of each species (*92*, *93*). To minimize gear selection bias (backpack/towed barge versus boat electrofishing), we restricted our analysis to wadeable stream samples collected via backpack or towed barge electrofishing. This was prudent because electrofishing methods are generally biased toward larger body sizes (*94*) and boat sampling is biased toward larger specimens than backpack sampling. When seining was used in addition to electrofishing in wadeable samples, we pooled data across sampling gears.

Each sampling location was manually cross-referenced to a discrete stream reach in the National Hydrography Dataset version 2.1 (*95*). Sample locations were visually cross-referenced to the nearest flow gage in a geographic information system (refer to the “Flow and environmental data” section). In this way, we identified flow records that were representative of hydrologic conditions at a given fish sampling site. We also used a relative drainage area criterion (upstream drainage area at a fish sample site must be within ±10% of a matched flow gage) to ensure meaningful pairings among sample sites and flow gages. Last, we did not permit a major discontinuity (a dam or large tributary) to occur between a sampling site and its representative flow gage. If these criteria could not be met for a given fish sample, then it was removed from the dataset.

Potential errors in the raw fish size data were detected and removed using length-weight regressions (refer to text S1). When repeat fish samples were collected from the same stream, we retained only the most recent sample. The “ritis” R package (*96*) was used to standardize species’ names and to screen any specimens lacking valid species-level identifications (primarily hybrids). The “fishtree” R package (*97*) was also used to screen specimens lacking complete phylogenetic information. The final fish dataset included 272 samples with paired flow gages across CONUS, with a mean drainage area of 4580 km^2^ (SD = 18,590).

For each of the sampled fish assemblages, maximum likelihood estimation was used to model the ISD and estimate *b* directly from the individual body mass data (*46*). Maximum likelihood is the preferred method to estimate *b*, as it avoids the use of binned mass data in linear regression models (*46*, *98*). We used the “sizeSpectra” R package *99*) with the negative log-likelihood method of Edwards *et al.* (*46*) to estimate *b*, following the example of Arranz *et al.* (*35*).

Trait data were obtained from multiple sources. Although many exhibit intraspecific plasticity or vary with ontogeny (e.g., trophic level and thermal tolerance) (*15*, *100*), we lacked information to assign intraspecific or stage-specific traits to individuals. We therefore used generalized species-level trait assignments, which broadly characterize adult behaviors. Trophic level was estimated for each species as the average value reported in FishBase (*48*), and critical thermal maximum was estimated from Comte and Olden (*47*). Species’ age at maturity, fecundity, spawning season, and reproductive mode were obtained from FishTraits (*33*). Missing values for continuous traits were imputed through ancestral reconstruction with the “Rphylopars” R package (*101*).

Each fish species was then oriented within a continuum of life three history strategy end points. Specifically, we used archetype analysis of a nonmetric multidimensional scaling ordination to assign each species an equilibrium-periodic-opportunistic affinity score (*102*). This ordination was constructed from a Gower dissimilarity matrix (*103*), using age at maturity, fecundity, spawning season, and parental care as inputs. Ordinal parental care values were recoded from reproductive mode as follows: nonguarder = 1, brood hider = 2, substrate chooser = 3, and guarders/bearers = 4. Recognizing that many species exhibit intermediate strategies, rather than a single life history strategy, we calculated abundance-weighted affinity scores for each of the three life history strategies. Assemblage-weighted affinity scores for the periodic and equilibrium end points were negatively correlated (*r* = −0.81, *P* < 0.05), so we retained only the periodic and opportunistic affinities for further analysis. Assemblage-weighted averages were also calculated for critical thermal maximum and trophic level for each sample.

### Flow and environmental data

We used a dataset of environmental flow metrics calculated from streamflow data obtained from US Geological Survey flow gages (*104*). We filtered this dataset to gages with ≥10 years of discharge records. The dataset contains a suite of metrics representing flow magnitude, frequency, duration, and the timing of both high- and low-flow events (defined, respectively, as the 90th and 10th percentile nonexceedance flows when normalized by drainage area). The “observed” value for each flow metric was compared with an “expected” value that represented flow in the absence of anthropogenic disturbance (*90*, *105*). Expected flow metrics were calculated following Carlisle *et al.* (*105*) and Eng *et al.* (*106*, *107*). Observed-to-expected (O/E) ratios were then used to estimate the size and direction of deviations from the natural flow regime (*104*). O/E ratios were provided for each flow metric at each gage on an annual basis, from 1980 to 2019.

Next, directionality was inferred for each O/E ratio. When an observed value was greater than the expected value after accounting for model error, it received an “inflated” designation (for magnitude, duration, and frequency metrics) or a “later” designation (for high- and low-flow timing metrics). When an observed value was less than the expected value after accounting for model error, it was designated “diminished” (magnitude, duration, and frequency metrics) or “earlier” (flow timing metrics). In cases where the O/E ratio was very small (observed value not substantially larger or smaller than expected value, after accounting for model error), an “indeterminate” label was assigned. For each of the flow metrics, we then calculated the proportion of years with inflated versus diminished conditions or earlier versus later conditions. These proportions were interpreted as measures of flow alteration severity that ranged from 0.0 to 1.0, with higher values indicating more frequently altered flow regimes. By using the proportions in subsequent analyses, we effectively standardized the flow data. This aided in interpretation and ensured the linear model assumptions of path analysis (refer to the “Path analysis” section) were met.

A subset of flow metrics was then selected to minimize collinearity (removing one variable from each pair of highly correlated variables using a threshold of |*r*| > 0.7) and enhance model parsimony. Flow duration metrics were first omitted because they were inversely related to frequency metrics (on an annual basis, more frequent flows are necessarily of shorter duration). We then selected one directional value from each of the three remaining flow metric classes (magnitude, frequency, and timing): (i) low-flow magnitude diminishment (higher values indicate more years with reduced low flows); (ii) high-flow frequency inflation (higher values indicate more years with elevated frequency of high flows); and (iii) later high flows (higher values indicate more years with delayed high flows).

Last, we included two land use metrics that are known to influence flow regimes and fish assemblages. Percentages of developed land (high- to medium-intensity land use) and crop land within the upstream drainage basin at each sampling site were downloaded from Wieczorek *et al.* (*108*). To account for natural variation in landscape characteristics across longitudinal and regional gradients, we added the upstream drainage area and ecoregion for each sample site as fixed effects to the path analysis model (refer to the “Path analysis” section). Ecoregions (aggregated level III ecoregions) (*109*) were further aggregated for sample size considerations into northern (Upper Midwest and Northern Appalachians), southern (Coastal Plains and Southern Appalachians), central (Southern, Temperate, and Northern Plains), and western (Western Mountains and Xeric) (fig. S4).

### Path analysis

Before modeling, data were transformed to improve normality (log*_x_* transformations or log_*x*+1_ transformations when zero values present) and standardized (*z*-transformed to mean = 0, SD = 1). Piecewise path analysis and stepwise model selection were then used to build a quantitative, system-level model of trait, flow, and land use effects on *b*, while controlling for the fixed effects of drainage area and ecoregion (hereafter, “control variables”). We began by selecting optimal fixed effects (all trait, flow, land use, and control variables) on *b* with a stepwise model selection process, using the Akaike information criterion (AIC) (*110*) to identify best-fitting models. When traits with significant effects on *b* were confirmed (*P* > 0.05), we again used AIC to detect fixed effects of flow, land use, and control variables on these traits. The process was repeated a third time to select fixed effects of land use and control variables on flow. Optimal models for *b*, traits, and flow were then combined in a single piecewise path model. In the combined model, bidirectional correlations between all pairs of trait and flow variables were included to account for potentially omitted covariates that may otherwise create residual correlations among endogenous variables. All modeled paths were linear regression models.

Path model fit was evaluated with Fisher’s *C* and the associated test for conditional independence, where a nonsignificant *P* value (>0.05) is evidence of appropriate model fit to the data (i.e., the model is independent of omitted paths). All path models were built with the “piecewiseSEM” R package (*111*). Standardized direct, indirect, and total (direct + indirect) effect sizes were calculated for each variable with the “semEff” R package (*112*).
