# Supplementary material for: Integrating theory and empirical patterns: Fish body size distributions, life history traits, and environmental flows in streams
Source: Sci Adv. 2025 Dec 19;11(51):eadu4026. doi: 10.1126/sciadv.adu4026 (PMC12716417; doi:10.1126/sciadv.adu4026)
Supplement: Supplementary file 1 — Text S1 Figs. S1 to S4 Tables S1 and S2 [file sciadv.adu4026_sm.pdf]

Science Advances
AAAS

Supplementary Materials for

Integrating theory and empirical patterns: Fish body size distributions, life history traits, and environmental flows in streams

Taylor Woods *et al.*

Corresponding author: Taylor Woods, tewoods@usgs.gov

Sci. Adv. **11**, eadu4026 (2025)
DOI: 10.1126/sciadv.adu4026

This PDF file includes:

Text S1
Figs. S1 to S4
Tables S1 and S2

Supplemental Text S1. Cleaning individual body size data.

We filtered fish assemblage data to mitigate potentially erroneous individual size measurements (fig. S1a). First, we removed individuals lacking total length (TL) or mass (M) information and those measured collectively (e.g., > 1 individual counted). We collected maximum body length (TL mm) data from the FishTraits database, supplemented with information from FishMorph when possible and removed records where reported TL > maximum TL in the trait database (fig. S1b). We also removed individuals with reported M ≤ 2.0 g to account for potential sampling biases in fish wetted weights at small sizes (fig. S1c). We performed species-specific length-mass regressions (LMR; among all sampling events) of *ln* TL on *ln* M and removed outlier values defined as observations with Cook's distance (D) > 4/n where n is the sample size for the regression (fig. S1d). We restricted our fish dataset to sampling event ×gear type combinations with ≥ 30 individuals (fig. S1e) and removed potential influential outlier records within these sampling events (among all individuals, regardless of taxonomy) based on Cook's D estimated from LMR using the methods described above (fig. S1f).

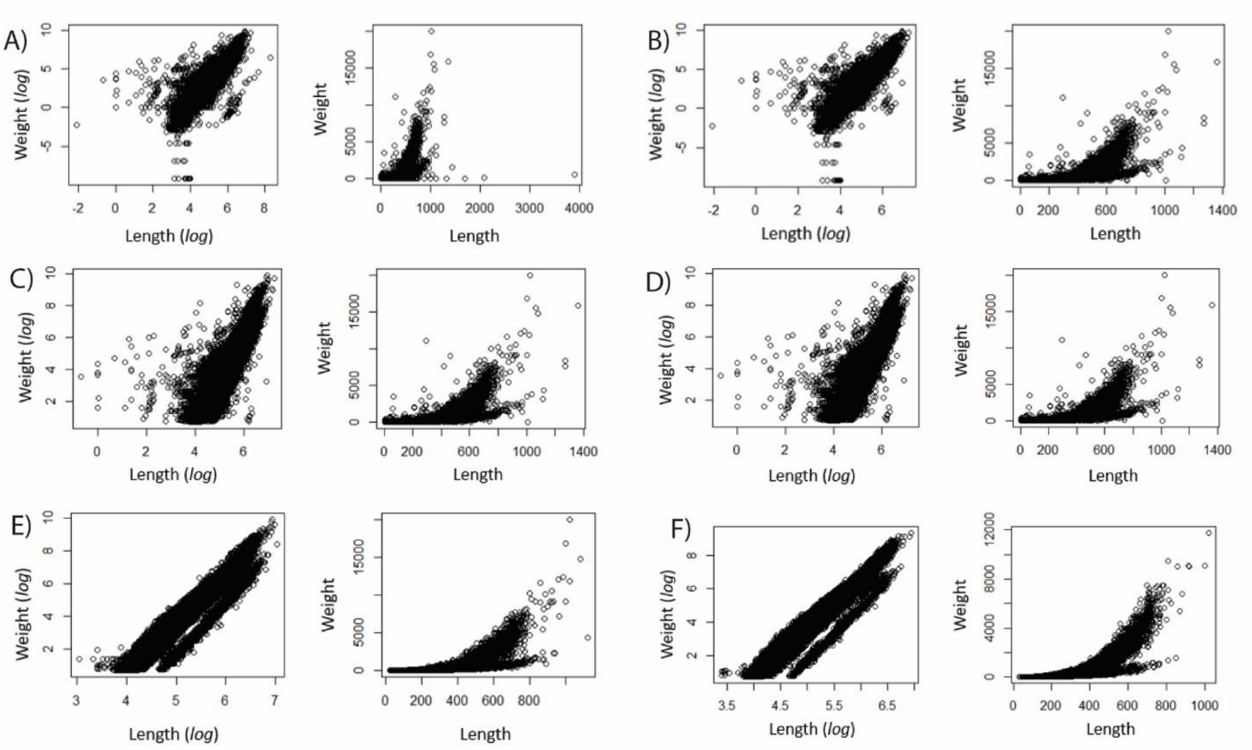

Figure S1. Filtering raw individual body size information. In all panels (a-f), left plots show length-mass regressions (LMR) of individual length (total length in mm; TL) and mass (weight in grams; M) on log-log (natural log) axes and right plots show untransformed data. Panels show: a) LMR from the raw dataset, prior to filtering for erroneous TL or M values, b) individual TL and M following filtering for maximum size, c) individual TL and M, omitting small individuals (< 2.0 grams M), d) individual TL and M, following species-specific length-mass regressions, e) individual TL and M after sample size filtering, and f) individual TL and M after removing outliers from sample-specific length-mass regressions. Panel f shows the filtered dataset that was used to build community size-spectra from individual mass measures.

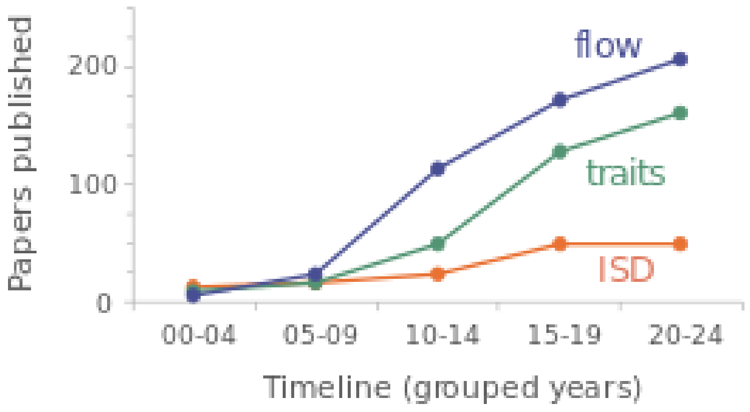

Figure S2. Literature trends through time for ISD, functional traits, and environmental flows. Figure shows publication rates (number of papers) through time for environmental flow (purple), traits (green), and individual size distributions (ISD) from 2000 – 2024. Publication rates were estimated between 2000-2024 with Web of Science keyword searches (https://www.webofscience.com/wos/), iterated over five-year time intervals (2000-2004, 2005-2009, etc.). All searches included the base prompts: fish AND (freshwater OR stream OR river OR lake). In addition to these base prompts, ISD research included the prompts: AND ("individual size distribution" OR "size spectrum" OR "size spectra"). Trait-based research included the base prompts plus: AND ("functional trait" OR "life history trait" OR "trait-based"). Environmental flows research included the base prompts plus: AND ("environmental flow" OR "e flow" OR "flow effect").

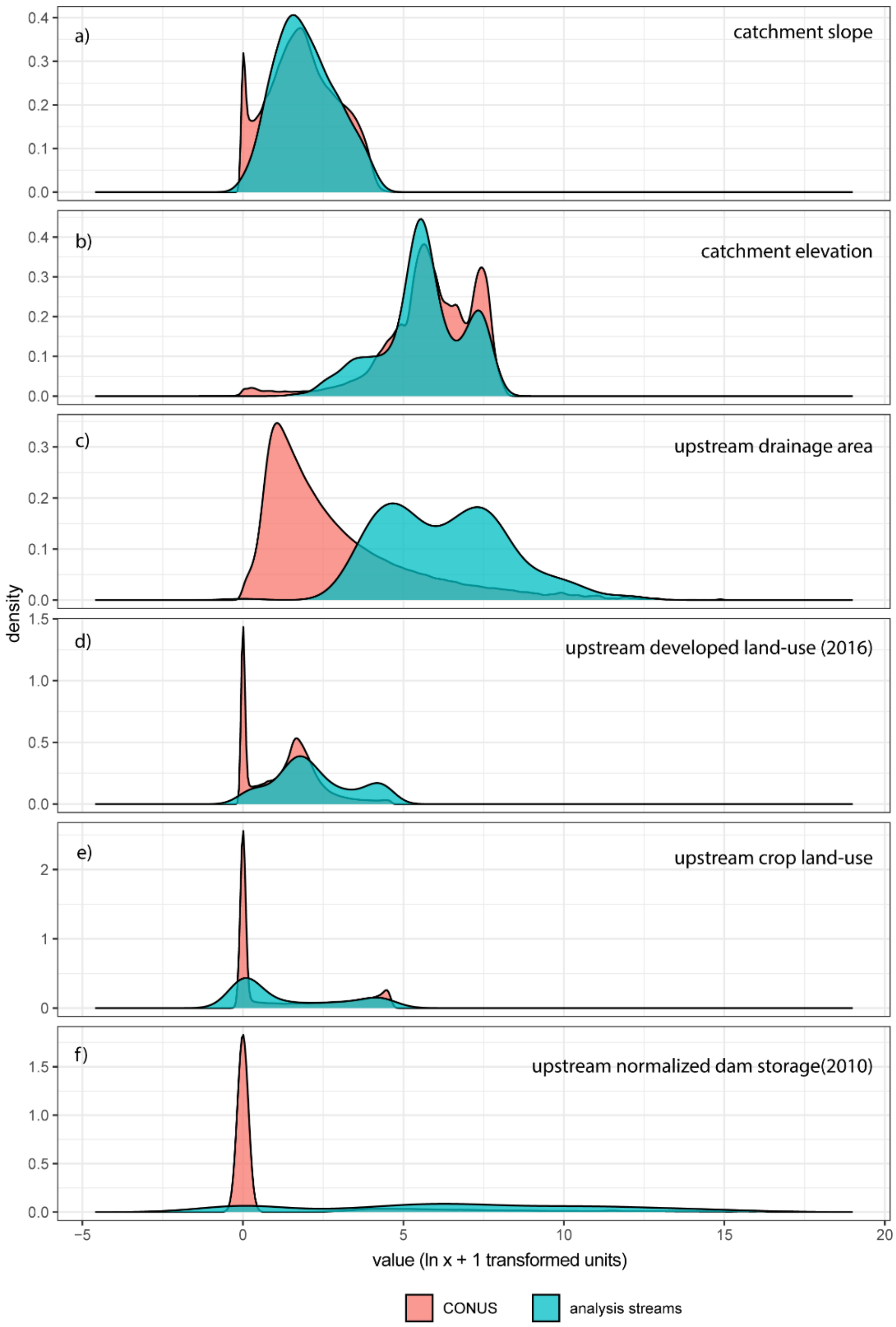

Figure S3. Distribution of characteristics at sample streams and all streams nationally. We show density plots for streams in our analysis (blue) compared to all streams in the Contiguous United States (CONUS) (pink) for a selection of natural (a-c, catchment slope, elevation, and upstream drainage area, respectively) and anthropogenic (d-f, upstream developed land-use and crop land-use from the 2016 National Land Cover Dataset, and upstream normalized dam storage) variables using data from Wieczorek et al (108). Values are $\ln_{x+1}$ transformed for all

variables and negative values of elevation were removed prior to plotting (only applicable for CONUS streams).

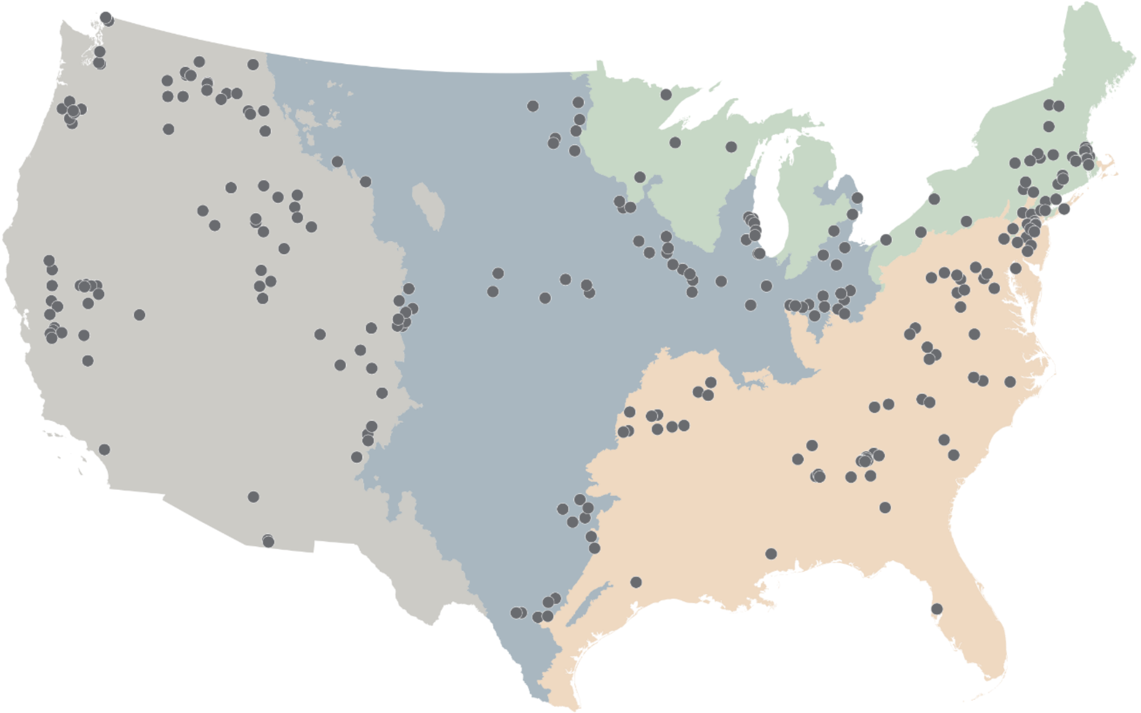

Figure S4. Map of regions and sampling sites. Regions are shown by colored polygons for the Southern (orange), Northern (green), Central (blue), and Western (grey). Paired gage and biological sampling sites retained in the analysis are represented by points. We obtained shapefiles from the Environmental Protection Agency at https://www.epa.gov/national-aquatic-resource-surveys/ecoregions-used-national-aquatic-resource-surveys.

Table S1. Potential mechanisms to account for hypothesized links in the meta-model. We describe hypothesized mechanisms of trait, flow, and land use effects on the exponent of the individual size distribution for each link indicated by letters on Fig. 1 (main text). References supporting each link are provided in the Materials and Methods.

Link	Hypothesized mechanism
A	Species' critical thermal maxima may be inversely related to the ISD *b* value (decreasing ISD *b*) because fish body size tends to decrease with temperature.
B	The periodic life history strategy favors large (increasing the ISD *b* value), highly fecund species with the ability to survive suboptimal conditions while awaiting suitable conditions for spawning events.
C	The opportunistic life history strategy favors small (decreasing the ISD *b* value), rapidly maturing species that use small clutch sizes and frequent spawning events to offset the effect of unpredictable/unstable environmental conditions.
D	Fish body size often increases (increasing the ISD *b* value) at higher trophic levels.
E	Non-advective (air-water) heat exchange and longer hydraulic resident times during low flow (drought) conditions may increase water temperature, exposing fishes to warmer conditions.
F	Low flow (drought) conditions may favor opportunistic species that use short generation times, specific egg deposition behaviors (e.g., deposition in crevices or adhesion to gravel), and multiple (small) clutches per season to maximize survival odds for some offspring.
G	Juvenile fishes may benefit from extended, periodic access to nursery habitat and food subsidies in floodplain habitats.
H	Repeated or extended high flow conditions may provide access to food subsidies in floodplain habitats, supporting rapid fish growth and abundant prey for large, high trophic level predators.
I	Frequent changes in the seasonal timing of high flow events, which can scour vulnerable eggs and juveniles, may favor opportunistic species that deposit multiple small clutches per year.
J	Seasonal mismatch between high flow timing and the most productive spring/summer growth period may negate the trophic benefits of floodplain subsidies for fishes.
K	Soil compaction (reduced groundwater infiltration) and irrigation withdrawal in crop fields may decrease low flow magnitude.
L	Soil compaction and reduced evapotranspiration in crop fields may increase the duration or frequency of high flow events.
M	Reduced evapotranspiration, altered soil permeability, irrigation dams, and seasonal irrigation in crop fields may influence the magnitude and timing of high flow events.
N	Rapid runoff from paved surfaces in developed areas may promote a flashy hydrograph with more frequent high flow events.
O	Altered runoff and water retention infrastructure in urban settings may accelerate or delay the timing of high flow events.

Table S2. Distribution of characteristics at sample streams and all streams nationally. We provide minimum (min), maximum (max), mean, and standard deviation (SD) for a selection of natural (catchment slope, elevation, and upstream drainage area) and anthropogenic (upstream developed land-use and crop land-use from the 2016 National Land Cover Dataset, and upstream normalized dam storage) variables at streams in our analysis (analysis) and all CONUS streams (indicated in the 'Type' column) using data from Wieczorek et al. (108).

**Variable**	**Type**	**Min**	**Max**	**Mean**	**SD**
Catchment slope	CONUS	0	370	9.94	12.2
	Analysis	0	63.5	10	11.7
Catchment elevation (m)	CONUS	-84.5	4096	674	705
	Analysis	4.8	2666	542	621
Upstream drainage area (square kilometers)	CONUS	0	3133387	3783	65374
	Analysis	0.01	210858	4548	18601
Upstream developed land-use	CONUS	0	100	6.19	12
	Analysis	0	99.4	18.7	26.1
Upstream crop land-use	CONUS	0	100	15.3	27
	Analysis	0	92.4	16.2	27.4
Upstream normalized dam storage (acre-feet)	CONUS	0	1.78E+08	233994	4061624
	Analysis	0	11331482	291853	1293191
